# Malignancy risk in AUS thyroid lesions: comparison between FNA and CNB with implications for NIFTP diagnosis

**DOI:** 10.3389/fendo.2025.1692071

**Published:** 2025-10-24

**Authors:** Yeseul Kim, Jae Ho Shin, You-Na Sung, Dawon Park, Harim Oh, Hyo Seon Ryu, Kyeong Jin Kim, Hyun Joo Kim, Sin Gon Kim, Hoon Yub Kim, Kwang Yoon Jung, Seung-Kuk Baek, Sangjeong Ahn

**Affiliations:** 1 Department of Pathology, Korea University College of Medicine, Anam Hospital, Seoul, Republic of Korea; 2 Department of Radiology, Korea University College of Medicine, Anam Hospital, Seoul, Republic of Korea; 3 Department of Surgery, Korea University Medical Center (KUMC) Thyroid Center, Korea University Hospital, Korea University College of Medicine, Seoul, Republic of Korea; 4 Division of Colon and Rectal Surgery, Department of Surgery, Korea University Hospital, Korea University College of Medicine, Seoul, Republic of Korea; 5 Division of Endocrinology and Metabolism, Department of Internal Medicine, Korea University College of Medicine, Seoul, Republic of Korea; 6 Department of Nuclear Medicine, Korea University College of Medicine, Anam Hospital, Seoul, Republic of Korea; 7 Department of Otorhinolaryngology-Head and Neck Surgery, Korea University College of Medicine, Anam Hospital, Seoul, Republic of Korea

**Keywords:** thyroid nodule, atypia of undetermined significance, fine-needle aspiration, core-needle biopsy, NIFTP

## Abstract

**Background:**

We aimed to evaluate and compare the efficacies and roles of core-needle biopsy (CNB) and repeat fine-needle aspiration (rFNA) in diagnosing thyroid nodules initially diagnosed as atypia of undetermined significance (AUS) by FNA. Additionally, we aimed to investigate the potential of CNB in diagnosing non-invasive follicular thyroid neoplasm with papillary-like nuclear features (NIFTP) and other follicular neoplasms (FNs), addressing its advantages over FNA in overcoming the diagnostic limitations of FNA.

**Methods:**

Overall, 635 nodules (7.3%) initially diagnosed as AUS were retrospectively reviewed from among 8,670 thyroid FNAs that were performed between 2018 and 2021. Malignancy rates were calculated as upper and lower limit estimates. rFNA was performed on 315 AUS nodules, and CNB was conducted on 62 patients.

**Results:**

Comparing the outcomes, CNB showed significantly fewer non-diagnostic results than rFNA (0% vs. 44.4%, p = 0.008) and a higher rate of FN diagnosis (11.3% vs. 0.3%, p < 0.001). In the AUS category, CNB demonstrated higher diagnostic rates for FNs, including NIFTP and follicular variant papillary thyroid carcinoma (50% vs. 18%). CNB significantly reduced the rate of insufficient diagnoses and increased the rate of diagnosing FNs. Moreover, CNB proved more effective than rFNA in diagnosing FNs, including NIFTP, within the AUS category, ensuring accurate detection without underdiagnosis.

**Conclusion:**

CNB may serve as a more reliable diagnostic tool for cases initially classified as AUS, particularly when repeat insufficient results are obtained or when diagnosing FNs and NIFTP.

## Highlights

- CNB appeared to reduce non-diagnostic rates in AUS thyroid nodules and tended to improve the detection of follicular neoplasms, including NIFTP.
- CNB may contribute to minimizing underdiagnosis and supporting more informed clinical management of AUS nodules.

## Introduction

Ultrasound (US)-guided fine-needle aspiration (FNA), a standard diagnostic modality for evaluating thyroid nodules, is cost-effective and safe (1, 2). Although FNA has a high diagnostic accuracy and is relatively simple, it has certain limitations, including a false-negative rate and a relatively high incidence of non-diagnostic or indeterminate results. The non-diagnostic rate is approximately 10%, and the diagnosis rate of atypia of undetermined significance (AUS) is up to 10%–20%, depending on the institution (3–7). Furthermore, a repeat FNA (rFNA) following an initial non-diagnostic result may yield a non-diagnostic outcome in up to 50% of cases. Moreover, FNA is known to have a lower diagnostic accuracy for both follicular lesions and medullary thyroid carcinoma, often leading to rFNAs or unnecessary surgeries (7–11). Therefore, additional diagnostic tools are needed to overcome these limitations and improve thyroid nodule evaluations.

Several studies have proposed core-needle biopsy (CNB) as an alternative or complementary tool to FNA (12–14). CNB is safe, well-tolerated, and associated with a low incidence of complications when performed by experienced operators (12, 15). Importantly, CNB has the potential to overcome some inherent limitations of FNA by obtaining larger tissue samples, thereby reducing non-diagnostic results due to scant follicular cells and enabling more detailed assessment of histological architecture and the tumor capsule. In addition, CNB specimens allow for immunohistochemical analysis, which may further aid in differential diagnosis. Previous studies have consistently reported that CNB yields lower rates of inconclusive results, including non-diagnostic or AUS, compared with repeat FNA in nodules with prior indeterminate cytology (13, 14, 16–18).

Since the introduction of non-invasive follicular thyroid neoplasm with papillary-like nuclear features (NIFTP) in 2017, it has been noted that NIFTP is typically diagnosed as Bethesda categories III, IV, and V on FNA. This creates challenges in definitively diagnosing NIFTP preoperatively. To address this challenge, several researchers have proposed distinct features of NIFTP that differentiate it from classic papillary thyroid carcinoma (PTC) on FNA (19–21). Strickland et al. (22) suggested that the presence of papillae, pseudoinclusions, or psammomatous calcifications indicates classic PTC, whereas NIFTP or invasive follicular variant PTC may be more likely when microfollicles predominate and papillae, pseudoinclusions, or psammomatous calcifications are absent. However, not all cases exhibit these features, and cytological diagnosis of NIFTP remains challenging. Because the diagnosis of NIFTP ultimately requires the evaluation of encapsulation, capsular and vascular invasion, and growth patterns—features that cannot be reliably assessed on cytology—core-needle biopsy may provide an advantage by preserving tissue architecture and enabling capsule assessment.

Therefore, we aimed to evaluate and compare the efficacies and roles of CNB and FNA in diagnosing thyroid nodules initially classified as AUS on FNA. Furthermore, we investigated the role of CNB in diagnosing NIFTP and other follicular neoplasms (FNs), offering a direct comparison with FNA to clarify its diagnostic advantages.

## Materials and methods

### Case selection

**Figure 1:**
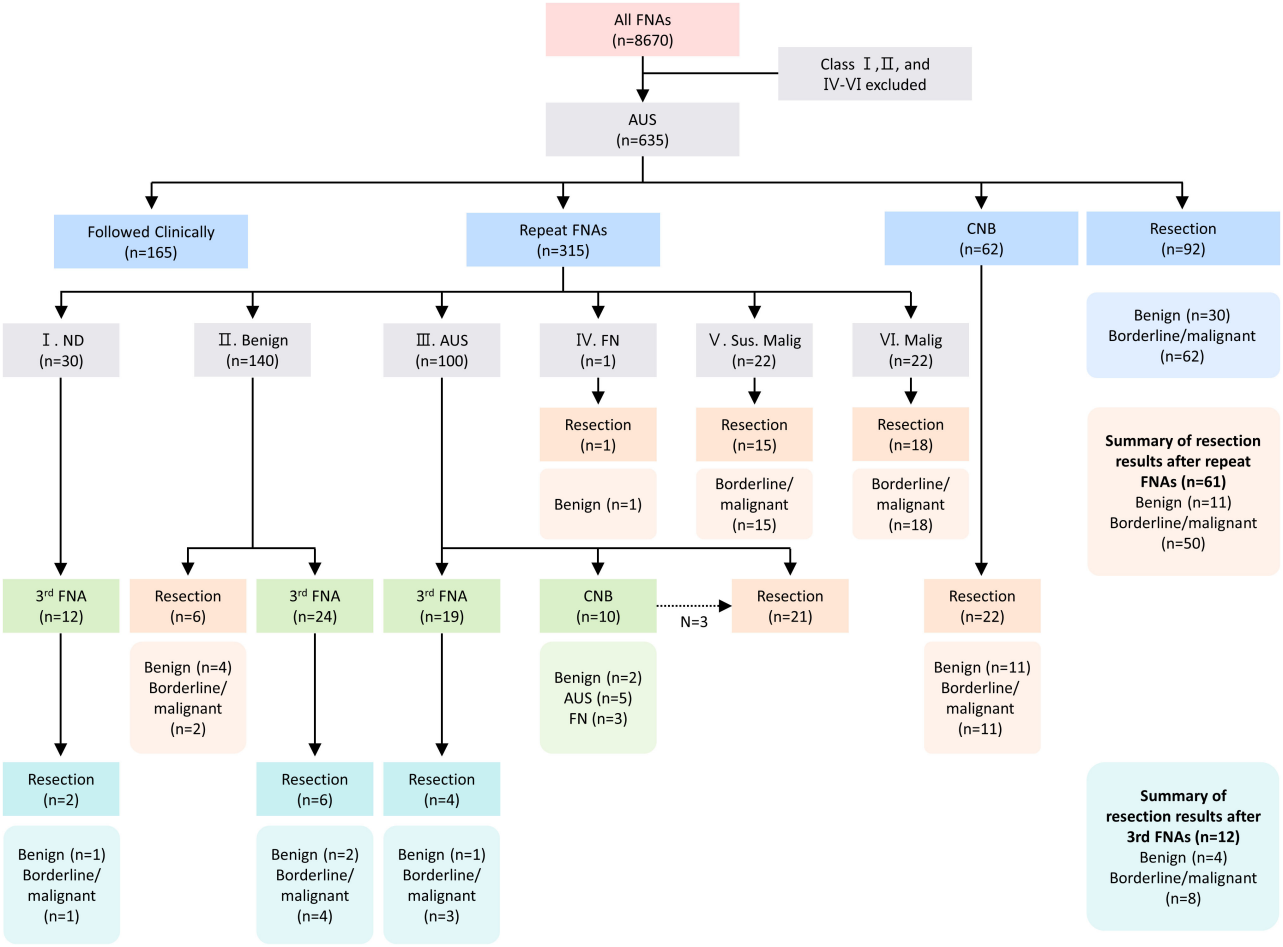
Flowchart of the patient population. AUS, atypia of undetermined significance; FNA, fine-needle aspiration; ND, non-diagnostic; FN, follicular neoplasm; Sus. Malig, suspicious for malignancy; Malig, malignant.

This study was approved by the Institutional Review Board (IRB) of Korea University Anam Hospital, where 8,670 FNA procedures were performed for thyroid nodules from January 2018 to December 2021 (**Figure 1**). Of the investigated nodules, 635 were classified as Bethesda class III and included in our analysis. In cases wherein patients had multiple nodules that underwent FNAs, one nodule was chosen based on larger size or more unfavorable US features. Among the groups with rFNA, thyroidectomy, and follow-up loss after being first diagnosed with AUS, the basal characteristics were compared to identify and minimize the selection bias.

Surgical management of AUS nodules followed the 2015 American Thyroid Association guidelines (1). Diagnostic surgical excision was performed in cases of persistent AUS on repeat FNA, inconclusive molecular testing, or the presence of worrisome clinical or sonographic features. Patient preference was also taken into account.

Repeat FNA and CNB were performed exclusively on mutually exclusive nodules that had been initially diagnosed as AUS on FNA; each nodule/patient was allocated to only one group without overlap.

The medical records of each patient were retrospectively reviewed for details such as the patients’ biological sex, age at the time of the first AUS cytopathology result, nodule size, and treatment pathway.

### US-guided FNA and CNB techniques

US-guided FNA and CNB were performed by two board-certified radiologists with >5 years of experience in head and neck imaging and thyroid intervention. US examinations were conducted utilizing a high-frequency linear probe with the following machines: EPIQ (Philips Healthcare, Bothell, WA, USA), Apolio (Canon, Tokyo, Japan), and HDI (Hitachi, Tokyo, Japan). Comprehensive US evaluations and risk stratification for thyroid nodules and cervical lymph nodes were conducted in accordance with the Korean Thyroid Imaging Reporting and Data System recommended by the Korean Society of Thyroid Radiology (23). Decision-making for performing FNA or CNB was based on the referring physicians’ and/or operators’ discretion. CNB was generally considered when the same nodule yielded non-diagnostic results on FNA more than once or when cytological findings were discordant with imaging findings.

The procedures were performed with the patients in the supine position, with their heads extended; contralateral neck rotations were occasionally performed for better visualization of the target nodule. Before the procedures, anatomical variations and important anatomical structures were carefully evaluated, especially in the vicinity of the needle trajectory. After activating the Doppler, vascular mappings were evaluated to gain access through the least vascular regions of the thyroid gland. For FNA, a 23-gauge needle was inserted via the trans-isthmic approach in parallel orientation so that the entire needle length was visualized on the US. Rapid to-and-fro motions were performed for capillary sampling, and gentle aspiration was performed to facilitate the sampling for liquid-based cytology (24). If scanty cellular debris was visualized in the liquid-based cytology bottle, additional FNA was performed. For CNB, lidocaine (1%–2%) was initially injected at the puncture site and near the thyroid capsule. Using an 18-gauge, spring-powered automatic device (TSK Ace-cut, Yokohama, Japan), the target nodule was biopsied via the trans-isthmic approach (12). The cutting cannula was positioned to encompass the intra-nodular component, nodular capsule, and the thyroid tissues (25). After the procedure, manual compression and ultrasound were performed to prevent procedure-related hemorrhages.

### Cytohistological analysis

FNA and CNB histology specimens were reviewed by two experienced pathologists (Kim Y. and Sung Y.-N.). FNA diagnoses were categorized into the following six categories according to the Bethesda System for Reporting Thyroid Cytopathology: I, non-diagnostic; II, benign; III, AUS; IV, FN; V, suspicious for malignancy; and VI, malignant (2). The diagnostic criteria for CNB have not been standardized for thyroid nodules; therefore, CNB histological diagnoses were categorized into the same six categories as those of the Bethesda System for Reporting Thyroid Cytopathology for the purpose of this study (26). All thyroid tissues were fixed in 10% neutralized formaldehyde. Nodules suspected of malignancy were totally embedded in paraffin and stained with hematoxylin and eosin. Histopathology was correlated to the nodule targeted by FNA by associating the location and size of the nodule. Incidental microcarcinoma was defined as a small papillary thyroid carcinoma that was not identified at the time of FNA but was subsequently detected only at thyroidectomy and was not concordant with the FNA-targeted index nodule. The presence of incidental microcarcinomas was noted but deemed benign for the purpose of this study. Tumors classified as borderline in the surgical specimens included NIFTP and follicular tumor of uncertain malignant potential (FT-UMP).

### Statistical analysis

The data are presented as the means and standard deviation for continuous variables and as the number of patients for categorical variables. Fisher’s exact test was used to compare categorical variables. All p*-*values <0.05 were considered to have statistical significance.

Estimates of the malignancy risk in thyroid nodules are subject to several biases because not all nodules undergo surgical resection or confirmatory diagnostic analysis. Therefore, we determined the conceivable ranges of the malignancy rates. The final malignancy rate was calculated as an upper limit estimate (ULE) and a lower limit estimate (LLE), as described by Ho et al. (27).

ULE was the malignancy rate of the resected nodules. LLE was calculated under the assumption that all the unresected nodules were benign and, therefore, the malignancy rate of all AUS cytopathology results (2).

## Results

### Patient characteristics

The study included 635 patients with a mean age of 56.0 ± 14.2 years. The male-to-female ratio was 0.28, and the mean nodule size was 14.8 ± 11.5 mm (**Table 1**). Among the 397 patients for whom ultrasound data were available, 35.8% were classified as Korean Thyroid Imaging Reporting and Data System (K-TIRADS) 5 and 45.6% as K-TIRADS 4, indicating that the majority of nodules were categorized as intermediate to high suspicion on ultrasound.

**Table 1:** Demographic data and characteristics of patients with atypia of undetermined significance nodules.

Characteristics	Total (N = 635)		
	No. of patients	rFNA (n = 315)	CNB (n = 62)
Sex			
Male	139 (21.9)	67 (21.3)	19 (30.6)
Female	496 (78.1)	248 (78.7)	43 (69.4)
Side			
Right	331 (52.1)	166 (52.7)	37 (59.7)
Left	282 (44.4)	137 (43.5)	24 (38.7)
Isthmus	22 (3.5)	12 (3.8)	1 (1.6)
Lymphocytic thyroiditis			
Negative	130 (20.0)	55 (17.5)	14 (22.6)
Positive	73 (11.0)	31 (9.8)	10 (16.1)
Not available	432 (69.0)	229 (72.7)	38 (61.3)
Resection			
Cases	188	73	22
Rate (%)	29.6	23.2	35.5
K-TIRADS category			
2	1 (0.1)	0(0)	0 (0)
3	73 (8.7)	45 (14.3)	3 (4.8)
4	181 (28.5)	79 (25.1)	33 (53.2)
5	142 (22.4)	67 (21.3)	6 (9.7)
Not available	238 (37.5)	124 (39.4)	20 (32.3)

FNA, fine-needle aspiration; CNB, core-needle biopsy; K-TIRADS, Korean Thyroid Imaging Reporting and Data System.

### Diagnostic outcomes of rFNA and CNB

Among the 635 nodules initially diagnosed as AUS, 50% (n = 315) underwent rFNA and 10% (n = 62) underwent CNB (**Figure 1**). In the rFNA group, 32% (n = 100) remained AUS, 44% (n = 140) were benign, 14% (n = 44) were suspicious/malignant, and 10% (n = 30) were non-diagnostic. A third FNA was performed in 55 cases, of which 22% (n = 12) underwent resection and 66% (n = 8) revealed borderline/malignant pathology.

**Table 2** presents a comparison between 315 rFNAs and 62 CNBs for nodules initially diagnosed with AUS. Among these, 59% were reclassified into definitive categories (benign, FN, suspicious for malignancy, or malignant), with benign being the most common diagnosis (44%). However, 41% of cases that underwent rFNA remained indeterminate, with AUS as the second most frequent outcome for all categories.

**Table 2:** Comparison of rFNA and CNB for the diagnosis of thyroid nodules with initial AUS.

Diagnosis ^a^(Bethesda System)	All (n = 377)		
	Repeat FNA (n = 315)	CNB (n = 62)	p-Value*
Non-diagnostic (I)	30 (9.5%)	0 (0%)	0.008
Benign (II)	140 (44.4%)	19 (30.6%)	0.049
AUS (III)	100 (31.7%)	31 (50.0%)	0.008
FN (IV)	1 (0.3%)	7 (11.3%)	<0.001
Suspicious for malignancy (V)	22 (7.0%)	1 (1.6%)	0.146
Malignant (VI)	22 (7.0%)	4 (6.5%)	1

FNA, fine-needle aspiration; CNB, core-needle biopsy; AUS, atypia of undetermined significance; FN, follicular neoplasm; rFNA, repeat fine-needle aspiration.

a Diagnosed according to six categories of the Bethesda System for Reporting Thyroid Cytopathology.

*Comparison of the diagnosis obtained using repeat FNA and CNB (Fisher’s exact test).

In the CNB group, 50% of nodules were reclassified into definitive categories, most commonly benign (31%), while the remaining 50% were diagnosed as AUS, making it the most frequent CNB outcome (**Table 2**). Unlike rFNA, CNB yielded no non-diagnostic results and showed significantly lower rates of non-diagnostic results (p = 0.008) and benign (p = 0.049) diagnoses and a higher rate of follicular neoplasm diagnoses (p < 0.001). Compared with rFNA, CNB yielded significantly higher AUS rates and a lower, although not statistically significant, rate of suspicious for malignancy (p = 0.146). Notably, CNB after an rFNA (n = 10) was conducted only when the rFNA also revealed AUS.

### Surgical outcomes and final diagnoses

Overall, 92 patients (14%) underwent direct thyroidectomy after the initial AUS diagnosis, with 66% (n = 62) diagnosed as borderline/malignant and 33% (n = 30) as benign. After rFNA, 19% (n = 61) underwent resection, of which 82% (n = 50) were borderline/malignant. In the CNB group, 35% (n = 22) underwent resection, with half (n = 11) showing borderline/malignant features.

For the patients who underwent surgery after rFNA (n = 72) and CNB (n = 22), the final diagnoses for the nodules are listed in **Table 3**. Detailed surgical outcomes stratified by ultrasound risk categories (K-TIRADS) in the rFNA and CNB groups are provided in **Supplementary Table 1**. A majority (62%) of malignant tumors were found in nodules diagnosed as AUS (31%) and malignant (31%) on rFNA. Similarly, a majority (81%) of malignant tumors were found in nodules diagnosed as AUS (45%) and malignant (36%) on CNB. Among the nodules diagnosed as benign by rFNA and subsequently operated on, 50% (6/12) were found to be malignant. In contrast, among those diagnosed as benign by CNB and subsequently operated on, 20% (1/5) were found to be malignant. For nodules initially diagnosed as AUS by rFNA, 75% (18/24) were confirmed as malignant after surgery, whereas for nodules initially diagnosed as AUS by CNB, the malignancy rate was 50% (5/10). All nodules initially diagnosed as FN by either rFNA or CNB were confirmed to be benign after operation. All nodules diagnosed as suspicious for malignancy or malignant by rFNA or CNB were confirmed as malignant on final surgical pathology. The final diagnosis of specimens that underwent surgery following an initial AUS diagnosis, along with their diagnostic categories by rFNA and CNB, are detailed in **Supplementary Table 2**.

**Table 3:** Results of rFNA and CNB in nodules initially diagnosed as AUS for which the final diagnoses were available.

Diagnosis ^a^(Bethesda system)	rFNA			CNB		
	Total (n = 72)	Benign (n = 14)	Malignancy (n = 58)	Total (n = 22)	Benign (n = 11)	Malignancy (n = 11)
Non-diagnostic (I)	2 (3%)	1 (7%)	1 (2%)	0	0	0
Benign (II)	12 (16%)	6 (43%)	6 (10%)	5 (23%)	4 (36%)	1 (9%)
AUS (III)	24 (34%)	6 (43%)	18 (31%)	10 (46%)	5 (45%)	5 (45%)
FN (IV)	1 (1%)	1 (7%)	0	2 (9%)	2 (18%)	0
Suspicious for malignancy (V)	15 (21%)	0	15 (26%)	1 (5%)	0	1 (9%)
Malignant (VI)	18 (25%)	0	18 (31%)	4 (18%)	0	4 (36%)

AUS, atypia of undetermined significance; CNB, core-needle biopsy; FN, follicular neoplasm; rFNA, repeat fine-needle aspiration.

a Diagnosed according to six categories of the Bethesda System for Reporting Thyroid Cytopathology.

Data are number of nodules, with percentage in parentheses.

NIFTP was included in the malignant category.

Cochran–Mantel–Haenszel p-value = 0.074.

Breslow–Day p-value = 0.846.

**Table 4** presents the risk of malignancy according to the two methods (risk of malignancy considering NIFTP as malignancy vs. risk of malignancy not considering NIFTP as malignancy).

**Table 4:** Implied risk of malignancy of rFNA and CNB.

Diagnosis ^a^(Bethesda system)	rFNA				CNB			
	Risk of malignancy if NIFTP ≠ CA (%)		Risk of malignancy if NIFTP = CA (%)		Risk of malignancy if NIFTP ≠ CA (%)		Risk of malignancy if NIFTP = CA (%)	
	Present	Literature (36)	Present	Literature (36)	Present	Literature (36),(43)	Present	Literature (36),(43)
Non-diagnostic (I)	3–50	6.4–59.3	3–50	6.4–59.3	N/A	13–70	N/A	13–70
Benign (II)	4–42	0.4–6.9	4–50	0.4–6.9	5–20	0.2–7.1	5–20	0.4–10.7
AUS (III)	14–56	24.6–70.8	18–72	26.1–75.0	10–30	6–27.3	16–50	6–35
FN (IV)	N/A	27–41	N/A	36–55	N/A	26.6–44.7	N/A	32.4–57.1
Suspicious for malignancy (V)	64–93	69–98	68–100	71–100	100	57–100	100	57–100
Malignant (VI)	82–100	73.5–100	82–100	73.5–100	100	100	100	100

AUS, atypia of undetermined significance; CNB, core-needle biopsy; FN, follicular neoplasm; rFNA, repeat fine-needle aspiration.

a Diagnosed according to six categories of the Bethesda System for Reporting Thyroid Cytopathology.

Data are number of nodules, with percentage in parentheses.

The diagnostic accuracy, sensitivity, specificity, positive predictive value, and negative predictive value are summarized in **Table 5**. The diagnostic accuracy for the rFNA and CNB groups was found to be 65.8% and 72.7%, respectively. The sensitivity for the rFNA and CNB groups was 56.9% and 45.5%, respectively, and the specificity was 100% for both groups.

**Table 5:** Diagnostic performance for malignancy of FNA and CNB in the surgical specimens.

Diagnostic performance	rFNA	CNB	p-Value
Diagnostic accuracy % (95% CI)	65.8 (54.3–75.6)	72.7 (51.8–86.8)	0.72
Sensitivity % (95% CI)	56.9 (44.1–68.8)	45.5 (21.3–72.0)	0.5
Specificity % (95% CI)	100 (79.6–100.0)	100 (74.1–100.0)	1
Positive predictive value % (95% CI)	100 (89.6–100.0)	100 (56.6–100.0)	1
Negative predictive value % (95% CI)	37.5 (24.2–53.0)	64.7 (41.3–82.7)	0.08

CNB, core-needle biopsy; rFNA, repeat fine-needle aspiration.

95% confidence intervals were calculated using the Wilson score method.

### Diagnosis of NIFTP and follicular variant papillary thyroid carcinoma in rFNA and CNB

**Figure 2:**
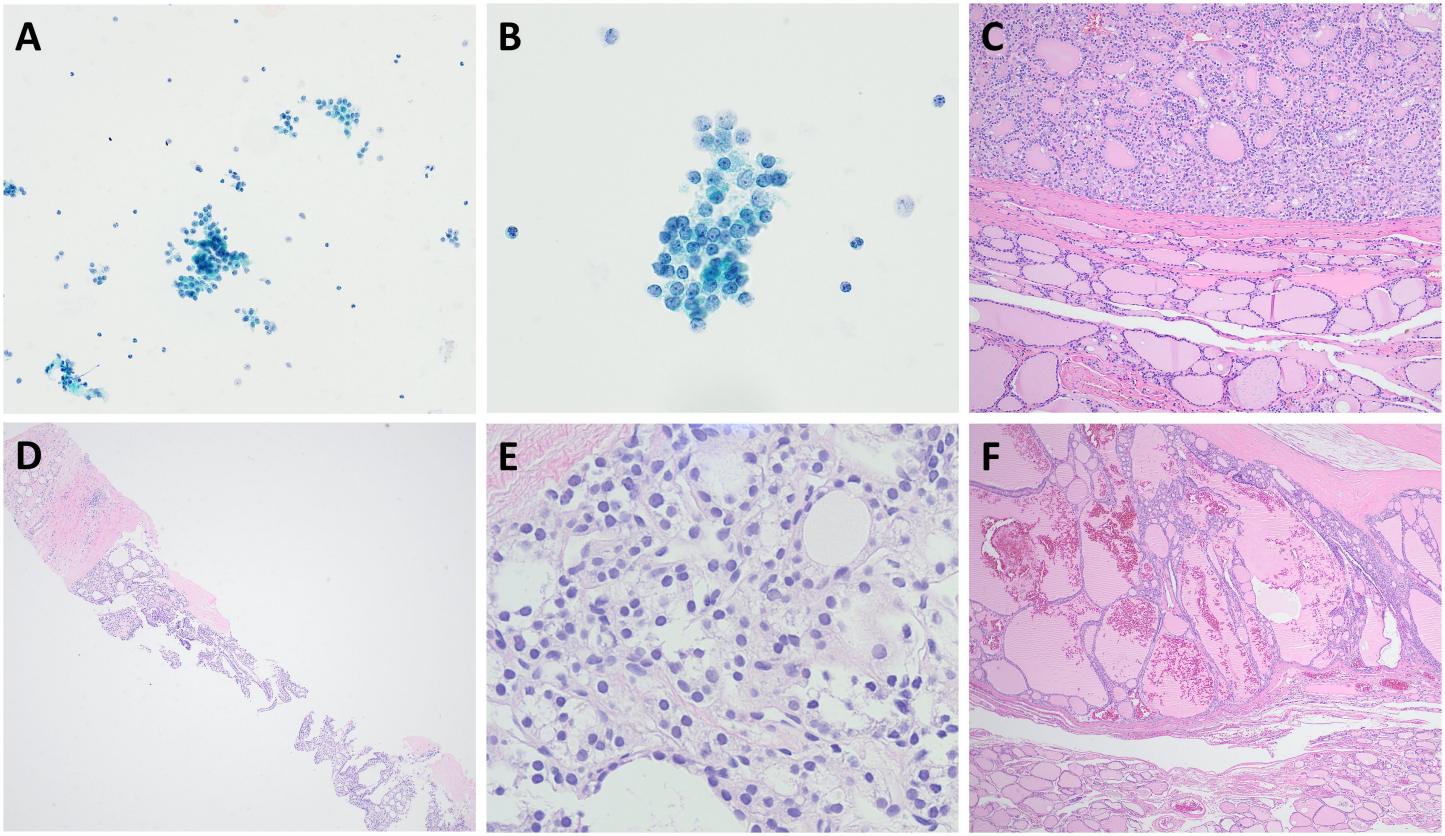
Cytological and histological features of non-invasive follicular thyroid neoplasm with papillary-like nuclear features (NIFTP) compared using repeat fine-needle aspiration (rFNA) and core-needle biopsy (CNB). **(A–C)** rFNA cytology and surgical resection specimen of NIFTP initially diagnosed as AUS, including the encapsulated mass. **(B)** rFNA showing follicular growth that is not clearly defined, with cells observed in clusters. Focal chromatin clearing and membrane irregularity can be seen; however, nuclear grooves or pseudoinclusions are not distinctly observed, corresponding to a nuclear score of 2. **(C)** The surgical specimen revealed a well-demarcated mass composed of neoplastic follicles of variable sizes. **(D–F)** CNB and surgical resection specimen of NIFTP initially diagnosed as AUS, including the thinly encapsulated mass. **(D)** The CNB specimen shows a thin capsule with macro- and microfollicular structures. **(E)** In high-power view, follicular growth and nuclear atypia can be observed, with membrane irregularity, increased size, and nuclear overlap, consistent with a nuclear score of 2. **(F)** The surgical specimen demonstrates a well-demarcated mass primarily composed of macrofollicular proliferation, with occasional microfollicles. Magnifications: **(A)** ×200; **(B)** ×400; **(C)** ×100; **(D)** ×40; **(E)** ×400; **(F)** ×100.

Among the AUS cases in the CNB group, 20% (n = 2) were diagnosed with follicular adenoma or oncocytic adenoma, and 50% (n = 5) were diagnosed with NIFTP or follicular variant papillary thyroid carcinoma (FVPTC). In comparison, in the rFNA group, 8% (n = 2) of AUS cases were diagnosed as follicular adenoma, and 18% (n = 7) were diagnosed with NIFTP or FVPTC (**Supplementary Table 2**). **Figure 2** compares the cytological and histological features of representative cases from rFNA and CNB in patients ultimately diagnosed with NIFTP through surgical resection. While rFNA cytology showed relatively low cellularity, the CNB cases exhibited higher cellularity as well as clearer identification of growth patterns and the nodule capsule.

## Discussion

FNA is a crucial, safe, and cost-effective procedure for diagnosing thyroid nodules (1, 2). However, it is limited to a cytomorphology examination and cannot provide information about the histological architecture or additional features (28, 29). Consequently, its diagnostic accuracy for rare thyroid malignancies, such as follicular carcinoma, lymphoma, undifferentiated carcinoma, medullary carcinoma, and metastases, is relatively low (30–34).

In contrast, CNB allows for a more detailed evaluation of the tissue architecture and enables immunohistochemical staining because a substantial amount of tissue can be obtained when performing this procedure. Due to its increased accuracy, CNB has been proposed as an alternative or complementary tool to FNA.

In this study, compared with the rFNA group, the CNB group had significantly fewer cases classified as Bethesda categories I and II and a higher proportion of cases classified as Bethesda categories III and IV. Previous studies have shown that compared with the rFNA group, the CNB group had a significantly lower rate of non-diagnostic (Bethesda category I) results and indeterminate results (Bethesda category III; 12.5%–40.9% in the CNB group vs. 45.3%–63% in the rFNA group) (13, 16, 17, 35, 36). Consistent with previous studies, the results of this study indicated that CNB was more effective than rFNA in reducing non-diagnostic results (rFNA 9.5% vs. CNB 0%).

Notably, CNB provides valuable information regarding the presence and thickness of the capsule, as well as the cellularity and architecture of FNs, which may aid in differentiating these lesions. In a study by Kim et al. (37), the rate of Bethesda category IV diagnosis using CNB was significantly higher (17.5%) compared with that with rFNA (2.0%), with similar findings reported by other studies (16, 37). In our study, the CNB group showed a higher proportion of cases diagnosed as NIFTP or FVPTC within the AUS category than did the rFNA group (50% vs. 18%). These findings suggest that CNB may be more effective than rFNA in diagnosing FNs, including NIFTP, within the AUS category, potentially improving detection while reducing underdiagnosis.

Furthermore, by more clearly identifying growth patterns and cytological atypia, CNB reduces the risk of over-diagnosing NIFTP or FVPTC as category IV or under-diagnosing them as category II. Instead, it increases the likelihood of appropriately classifying these lesions within categories III, IV, and V, thereby improving predictive accuracy for NIFTP and FVPTC (20, 38, 39). Additionally, the use of immunohistochemical markers such as HBME-1 and CD56 with CNB offers advantages in distinguishing benign from malignant tumors, further enhancing diagnostic precision (40, 41).

Among the patients diagnosed with Bethesda categories V and VI via rFNA and who underwent surgery (n = 33), 32 were diagnosed with PTC, including four cases of FVPTC. Additionally, of the 25 patients diagnosed with AUS in the rFNA group and who underwent surgery, 11 were found to have PTC, including six cases of FVPTC. In contrast, in the CNB group, among the patients diagnosed with Bethesda categories V and VI and who underwent surgery, four patients were diagnosed with PTC. This suggests that CNB is more definitive in diagnosing PTC in patients with a Bethesda category V or VI. Among the 10 patients diagnosed with AUS via CNB who underwent surgery, none were diagnosed with classic PTC. Furthermore, the negative predictive value of CNB was higher than that of rFNA in lesions that were initially diagnosed as benign and later confirmed to be benign after surgery (37.5% vs. 64.7%).

CNB allows clear visualization of the cellular clusters and the tumor’s surrounding microenvironment, including changes such as fibrosis and calcification; additionally, it allows definitive identification of follicular architecture. Therefore, CNB is expected to perform better than FNA in distinguishing between papillary and follicular lesions. These findings are consistent with those of previous studies. For example, Kim et al. (37) reported that CNB was associated with a reduced rate of indeterminate results and a higher diagnostic rate for patients with Bethesda category IV than was FNA. Further, Lee et al. (42) demonstrated in their validation study that CNB was an effective method for diagnosing FNs during thyroid nodule screening. Yoon et al. (43) found that compared with FN, CNB revealed significantly lower false-positive rates and a reduced rate of unnecessary surgeries. In line with these previous studies (16, 17, 37), our study also revealed that CNB revealed a higher diagnostic rate for Bethesda category IV than did rFNA (11.3% vs. 0.3%). The enhanced diagnostic accuracy of CNB for FNs can be attributed to its superior ability to evaluate the nodule capsule and growth patterns, compared with that of rFNA.

Consistent with prior literature (44, 45), our data also showed that nodules with high-suspicion ultrasound features (K-TIRADS 5) were frequently confirmed as malignant, suggesting that rFNA is most appropriate in this subset. In contrast, nodules with low- to intermediate-suspicion features (K-TIRADS 3–4) often remained indeterminate, where CNB provided additional value for differentiating FN and NIFTP.

**Figure 3:**
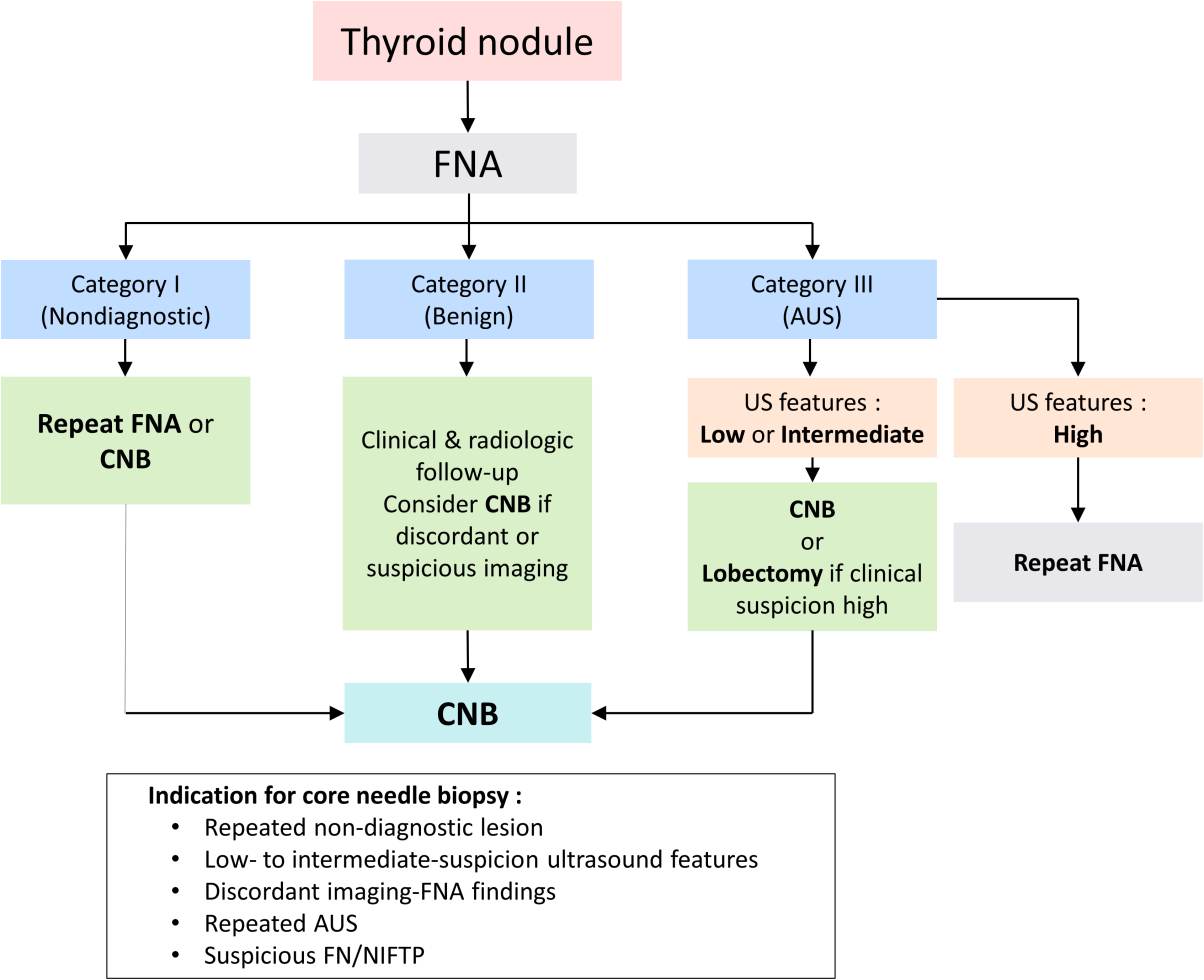
Proposed clinical diagnostic algorithm for the selective use of CNB. CNB, core-needle biopsy.

Based on our findings, we propose a clinical diagnostic flow incorporating CNB as a second-line tool in defined clinical contexts, including NIFTP. In particular, it proved useful in cases with repeat non-diagnostic results, low- to intermediate-suspicion ultrasound features, discordant imaging and cytology findings, persistent AUS, or suspected FN/NIFTP. As illustrated in **Figure 3**, this flowchart reflects a more refined approach to thyroid nodule evaluation, supporting the selective use of CNB to enhance diagnostic accuracy and reduce unnecessary surgeries.

This study had some limitations. First, as a retrospective study, not all thyroid nodules were evaluated with both rFNA and CNB, potentially introducing selection bias. In particular, the number of AUS nodules that underwent CNB was limited and substantially lower compared with the number of AUS nodules that underwent rFNA, which may have influenced the comparability of outcomes between the two groups. Additionally, the retrospective design limited control over potential confounders, including clinician decision-making, operator variability, and incomplete clinical or ultrasonographic data. Moreover, CNB was more often performed in nodules with intermediate-suspicion US features, reflecting a selection bias that may have contributed to the higher frequency of Bethesda III and IV results in this group. Furthermore, not all nodules underwent surgical excision for definitive histologic confirmation, which may have introduced additional selection bias. However, this reflects real-world clinical practice, where surgical confirmation is typically reserved for nodules with concerning cytological or imaging findings. Second, CNB diagnoses were categorized according to the Korean Thyroid Association’s 2019 Practice Guidelines for Thyroid Core Needle Biopsy (26). While the KTA guideline and the Bethesda System are not identical, they are closely aligned in their conceptual framework, which suggests that the potential for misclassification is relatively low. Third, the subclassifications of AUS were not incorporated into our analysis. Recent literature has identified distinct features and variable malignancy risks across AUS subcategories, indicating that the diagnostic utility of rFNA and CNB may differ within these groups. Future investigations specifically targeting these AUS subclassifications are warranted to address this limitation and provide further insights.

In conclusion, CNB appeared to provide more definitive results for nodules in Bethesda category I and tended to identify Bethesda category IV diagnoses more frequently than rFNA. Furthermore, CNB appeared to have higher accuracy, positive predictive value, and negative predictive value, which may help reduce unnecessary surgeries. Moreover, CNB may be more effective than rFNA in diagnosing FNs, including NIFTP, within the AUS category, suggesting a potential role in improving diagnostic precision. Overall, CNB may serve as a useful adjunctive diagnostic tool for cases initially diagnosed as AUS, particularly when repeat insufficient results are obtained or when evaluating follicular-patterned lesions such as FNs and NIFTP.

## Data Availability

The raw data supporting the conclusions of this article will be made available by the authors, without undue reservation.
