# Correction: Malignancy risk in AUS thyroid lesions: comparison between FNA and CNB with implications for NIFTP diagnosis

**DOI:** 10.3389/fendo.2025.1753907

**Published:** 2025-12-05

**Authors:** Yeseul Kim, Jae Ho Shin, You-Na Sung, Dawon Park, Harim Oh, Hyo Seon Ryu, Kyeong Jin Kim, Hyun Joo Kim, Sin Gon Kim, Hoon Yub Kim, Kwang Yoon Jung, Seung-Kuk Baek, Sangjeong Ahn

**Affiliations:** 1 Department of Pathology, Korea University College of Medicine, Anam Hospital, Seoul, Republic of Korea; 2 Department of Radiology, Korea University College of Medicine, Anam Hospital, Seoul, Republic of Korea; 3 Department of Surgery, Korea University Medical Center (KUMC) Thyroid Center, Korea University Hospital, Korea University College of Medicine, Seoul, Republic of Korea; 4 Division of Colon and Rectal Surgery, Department of Surgery, Korea University Hospital, Korea University College of Medicine, Seoul, Republic of Korea; 5 Division of Endocrinology and Metabolism, Department of Internal Medicine, Korea University College of Medicine, Seoul, Republic of Korea; 6 Department of Nuclear Medicine, Korea University College of Medicine, Anam Hospital, Seoul, Republic of Korea; 7 Department of Otorhinolaryngology-Head and Neck Surgery, Korea University College of Medicine, Anam Hospital, Seoul, Republic of Korea

**Keywords:** thyroid nodule, atypia of undetermined significance, fine-needle aspiration, core-needle biopsy, NIFTP

The funder “National Research Foundation of Korea (NRF) grant funded by the Korea government (MSIT), (No. RS-2025-02215813)” to Sangjeong Ahn was erroneously omitted. The original version of this article has been updated.

