# Supplementary material for: Malignancy risk in AUS thyroid lesions: comparison between FNA and CNB with implications for NIFTP diagnosis
Source: Front Endocrinol (Lausanne). 2025 Oct 24;16:1692071. doi: 10.3389/fendo.2025.1692071 (PMC12591977; doi:10.3389/fendo.2025.1692071)
Supplement: Supplementary file 1 [file Table1.docx]

| **Online Resource 1.** Surgical outcomes stratified by K-TIRADS categories in AUS nodules evaluated with rFNA and CNB | | | | | | | |
| --- | --- | --- | --- | --- | --- | --- | --- |
| **K-TIRADS** | **rFNA** | | | **CNB** | | | |
|  | Total  (n = 35) | Benign  (n = 6) | Malignancy  (n = 29) | | Total  (n = 13) | Benign  (n = 7) | Malignancy  (n = 6) |
| 3 | 8 (43.8%) | 2 (33.3%) | 6 (20.7%) | | 0 (0) | 0 (0) | 0 (0) |
| 4 | 10 (28.6%) | 3 (50.0%) | 7 (24.1%) | | 11 (84.6%) | 6 (85.7%) | 5 (83.3%) |
| 5 | 17 (48.6%) | 1 (16.7%) | 16 (55.2%) | | 2 (15.4%) | 1 (14.3%) | 1 (16.7%) |
| Abbreviations: CNB = core needle biopsy, rFNA = repeat fine-needle aspiration | | | | | | | |
