# Supplementary material for: Malignancy risk in AUS thyroid lesions: comparison between FNA and CNB with implications for NIFTP diagnosis
Source: Front Endocrinol (Lausanne). 2025 Oct 24;16:1692071. doi: 10.3389/fendo.2025.1692071 (PMC12591977; doi:10.3389/fendo.2025.1692071)
Supplement: Supplementary file 2 [file Table2.docx]

| **Online Resource 2.** Final Diagnosis of Specimens that Underwent Surgery Following an Initial AUS Diagnosis and Their Diagnostic Categories by rFNA | | | | | | | | | | | | | | | |
| --- | --- | --- | --- | --- | --- | --- | --- | --- | --- | --- | --- | --- | --- | --- | --- |
| **Histopathology** | **Total**  **(n=188)** | **Direct Op.**  **(n=92)** | **rFNA (n=315)** | | | | | | | **CNB after 1^st^ FNA (n=62)** | | | | | |
|  |  |  | **Op. after rFNA**  **total (n=73)** | **Diagnosis *(Bethesda system) (operation/rFNA)** | | | | | | **Op. after Biopsy**  **total (n=22)** | **Diagnosis *(Bethesda system) (operation/CNB)** | | | | |
|  |  |  |  | **I. ND**  **(n=2/30)** | **II. Benign**  **(n=12/140)** | **III. AUS**  **(n=25/100)** | **IV. FN**  **(n=1/1)** | **V. S/f Malig**  **(n=15/22)** | **VI. Malig**  **(n=18/22)** |  | **II. Benign**  **(n=5/19)** | **III. AUS (n=10/31)** | **IV. FN**  **(n=2/7)** | **V. S/f malig**  **(n=1/1)** | **VI. Malig**  **(n=4/4)** |
| TFND | 36 (19.3%) | 22 (23.9%) | 8 (11%) | 0 (0%) | 3 (25%) | 5 (20%) | 0 (0%) | 0 (0%) | 0 (0%) | 6 (27.2%) | 3 (60%) | 3 (30%) | 0 (0%) | 0 (0%) | 0 (0%) |
| LT | 1 (0.5%) | 1 (1.1%) | 0 (0%) | 0 (0%) | 0 (0%) | 0 | 0 (0%) | 0 (0%) | 0 (0%) | 0 (0%) | 0 (0%) | 0 (0%) | 0 (0%) | 0 (0%) | 0 (0%) |
| FA | 15 (8%) | 7 (7.6%) | 5 (6.8%) | 1 (50%) | 1 (8.3%) | 2 (8%) | 1 (100%) | 0 (0%) | 0 (0%) | 3 (13.6%) | 0 (0%) | 1 (10%) | 2 (100%) | 0 (0%) | 0 (0%) |
| OA | 4 (2.1%) | 0 (0%) | 2 (2.7%) | 0 (0%) | 2 (16.7%) | 0 (0%) | 0 (0%) | 0 (0%) | 0 (0%) | 2 (9.1%) | 1 (20%) | 1 (10%) | 0 (0%) | 0 (0%) | 0 (0%) |
| NIFTP | 12 (6.4%) | 4 (4.3%) | 6 (8.2%) | 0 (0%) | 1 (8.3%) | 4 (16%) | 0 (0%) | 1 (6.7%) | 0 (0%) | 2 (9.1%) | 0 (0%) | 2 (20%) | 0 (0%) | 0 (0%) | 0 (0%) |
| FT-UMP | 1 (0.5%) | 1 (1.1%) | 0 (0%) | 0 (0%) | 0 (0%) | 0 (0%) | 0 (0%) | 0 (0%) | 0 (0%) | 0 (0%) | 0 (0%) | 0 (0%) | 0 (0%) | 0 (0%) | 0 (0%) |
| FVPTC | 19 (10.2%) | 6 (6.5%) | 8 (11%) | 0 (0%) | 1 (8.3%) | 3 (12%) | 0 (0%) | 1 (6.7%) | 3 (16.7%) | 5 (22.7%) | 1 (20%) | 3 (30%) | 0 (0%) | 1 (100%) | 0 (0%) |
| Classic PTC | 86 (46%) | 46 (50%) | 36 (49.3%) | 0 (0%) | 3 (25%) | 7 (28%) | 0 (0%) | 13 (86.7%) | 13 (72.2%) | 4 (18.2%) | 0 (0%) | 0 (0%) | 0 (0%) | 0 (0%) | 4 (100%) |
| PTC with solid/trabecular | 1 (0.5%) | 0 (0%) | 1 (1.4%) | 0 (0%) | 0 (0%) | 1 (4%) | 0 (0%) | 0 (0%) | 0 (0%) | 0 (0%) | 0 (0%) | 0 (0%) | 0 (0%) | 0 (0%) | 0 (0%) |
| Anaplastic focus in NH | 1 (0.5%) | 1 (1.1%) | 0 (0%) | 0 (0%) | 0 (0%) | 0 | 0 (0%) | 0 (0%) | 0 (0%) | 0 (0%) | 0 (0%) | 0 (0%) | 0 (0%) | 0 (0%) | 0 (0%) |
| FTC | 7 (3.7%) | 3 (3.3%) | 4 (5.5%) | 0 (0%) | 1 (8.3%) | 3 (12%) | 0 (0%) | 0 (0%) | 0 (0%) | 0 (0%) | 0 (0%) | 0 (0%) | 0 (0%) | 0 (0%) | 0 (0%) |
| MTC | 2 (1.1%) | 0 (0%) | 2 (2.7%) | 0 (0%) | 0 (0%) | 0 | 0 (0%) | 0 (0%) | 2 (11.1%) | 0 (0%) | 0 (0%) | 0 (0%) | 0 (0%) | 0 (0%) | 0 (0%) |
| MALT lymphoma | 1 (0.5%) | 0 (0%) | 1 (1.4%) | 1 (50%) | 0 (0%) | 0 | 0 (0%) | 0 (0%) | 0 (0%) | 0 (0%) | 0 (0%) | 0 (0%) | 0 (0%) | 0 (0%) | 0 (0%) |
| DLBCL | 2 (1.1%) | 2 (.2%) | 0 (0%) | 0 (0%) | 0 (0%) | 0 | 0 (0%) | 0 (0%) | 0 (0%) | 0 (0%) | 0 (0%) | 0 (0%) | 0 (0%) | 0 (0%) | 0 (0%) |
| **Abbreviations: AUS* atypia of undetermined significance, *Classic-PTC* classic or not specified variant of papillary thyroid carcinoma, *CNB* core-needle biopsy, *DLBCL* Diffuse large B-cell lymphoma, FA follicular adenoma, *FN* follicular neoplasm, *FNA* fine-needle aspiration *FTC* follicular thyroid carcinoma, *FT-UMP* Follicular tumor of unmalignant potential, *FVPTC* follicular variant of papillary thyroid carcinoma, *LT* lymphocytic thyroiditis, *Malig* Malignant *MALT lymphoma* mucosa-associated lymphoid tissue lymphoma, *MTC* medullary thyroid carcinoma, *NH* nodular hyperplasia, *NIFTP* non-invasive follicular thyroid neoplasm with papillary-like nuclear features, *OA* oncocytic adenoma, *OP* operation, *S/f Malig* suspicious for malignancy, *TFND* thyroid follicular nodular disease | | | | | | | | | | | | | | | |
